# How Uncertainty Influences Lay People’s Attitudes and Risk Perceptions Concerning Predictive Genetic Testing and Risk Communication

**DOI:** 10.3389/fgene.2019.00380

**Published:** 2019-04-26

**Authors:** Sabine Wöhlke, Manuel Schaper, Silke Schicktanz

**Affiliations:** Department of Medical Ethics and History of Medicine, University Medical Center Göttingen, Göttingen, Germany

**Keywords:** genetic risk information, lay attitudes, focus group discussion, predictive genetic diagnostics, genetic test value, ethics, epistemic uncertainty, aleatoric uncertainty

## Abstract

The interpretation of genetic information in clinical settings raises moral issues about adequate risk communication and individual responsibility about one’s health behavior. However, it is not well-known what role numeric probabilities and/or the conception of disease and genetics play in the lay understanding of predictive genetic diagnostics. This is an important question because lay understanding of genetic risk information might have particular implications for self-responsibility of the patients.

**Aim:** Analysis of lay attitudes and risk perceptions of German lay people on genetic testing with a special focus on how they deal with the numerical information.

**Methods:** We conducted and analyzed seven focus group discussions (FG) with lay people (*n* = 43).

**Results:** Our participants showed a positive attitude toward predictive genetic testing. We identified four main topics: (1) Anumeric risk instead of statistical information; (2) Treatment options as a factor for risk evaluation; (3) Epistemic and aleatory uncertainty as moral criticism; (4) Ambivalence as a sign of uncertainty.

**Conclusion:** For lay people, risk information, including the statistical numeric part, is perceived as highly normatively charged, often as an emotionally significant threat. It seems necessary to provide lay people with a deeper understanding of risk information and of the limitations of genetic knowledge with respect to one’s own health responsibility.

## Introduction

Technological progress over the recent years has led to an unprecedented increase in speed and precision of genetic testing. The number of inherited disorders and risk factors that can be detected through genetic testing is growing rapidly and genetic testing has become almost a common component of routine medical care (Lerman et al., 2002). Diagnostically conclusive predictive genetic test results increasingly affect patients’ perceived responsibility for their health (e.g., Patenaude et al., 2002; D’Agincourt-Canning, 2006; Schicktanz, 2018). Predictive genetic testing – in contrast to diagnostic tests – aims at the risk assessment of how likely it is to develop a particular disease (especially a multifactorial disease) in the following years based on a statistical, probabilistic analysis (Genetic Alliance, 2009).

Current studies have examined challenges for lay people in dealing with risk information from genetic testing (Engelhardt et al., 2017; Han et al., 2017; Solomon et al., 2017).^1^ They showed that perceived risk is an important subjective psychological phenomenon that is closely intertwined with individual judgments about susceptibility to getting ill as well as potential benefits from interventions (Weinstein and Klein, 1995). Apart from these observations, professional standards expect doctors and geneticists to provide correct, unbiased and full information to patients based on the moral assumption of respecting patient’s autonomy (European Commission, 2004; UNESCO, 2004; European Society of Human Genetics, 2015). However, important ethical issues remain unsolved, such as how to ensure the right not to know, how to consider different models of informed consent in light of the predictive, probabilistic character of genetic risk information, or how to balance the interests of a person with the interests of their genetic relatives (Andorno, 2004; Beskow and Burke, 2010; Borry et al., 2014; Leefmann et al., 2017; Falahee et al., 2018; Inthorn, 2018). For lay people, these ethical issues complicate decision-making, since conflicting interests may occur in genetic testing. Therefore, laypeople may be ambivalent regarding the “right” decision, e.g., between not wanting to know about being affected by and also not wanting to pass on a monogenic disorder (Engelhardt et al., 2017). However, the focus of studies with lay people has so far mostly been on the mathematical or numerical understanding of probabilistic test results (Schapira et al., 2001; Gigerenzer and Gray, 2011). Currently, little is known about the influence of the moral dimension of decision-making based on the complexity of probabilistic test results. We assume that genetic risk information leads to complex decision-making and that this decision-making has a moral dimension for the individual and emerges in a specific social and cultural embedding.

Predictive genetic testing is scientifically framed as “risk information which is ethically relevant in the medical context, as there is rarely a thorough knowledge of probabilities assigned to the different diagnostic outcomes” (Palmboom and Willems, 2010; see also Brahier, 2012). Studies show that public awareness of genetic tests is high and that lay people believe that knowledge about the genetic background of diseases helps people to live longer (Henneman et al., 2013). Little is known about lay people’s perspectives on the values of predictive genetic tests and the information they give. Lay perspectives have gained importance since genetic testing is being used increasingly in different contexts of medicine and is also increasingly available directly to consumers over the internet (thus affecting more and more people) (Harvey et al., 2012). This availability is accompanied by a prevention discourse framing knowledge of genetic risks as an incentive for responsible health behavior. It is also important to include lay perspectives into ethical debates for reasons of epistemic justice (Schicktanz and Schweda, 2012). This study explored attitudes and experiences of lay people regarding the potential use of risk information for screening or treatment decisions, as well as their perceptions of the moral dimension of decision-making based on genetic risk information. The aim of our project is to explore the less well-known attitudes and risk perceptions of German lay people on genetic testing with a special focus on how they deal with the numerical information. After introducing background theories about understandings of genetic risk and related concepts of uncertainty, we present the results of a larger qualitative study we conducted. The perspective of lay people might be particularly helpful to improve general health communication as well as to assess the motivation to seek, and public acceptance of, genetic testing services. Our analysis shows how lay people deal with numerical probabilistic information, how it affects their potential decision and how they express moral uncertainty. Finally, we discuss the relevance of these attitudes toward, and experiences with, predictive genetic testing from an ethical and practical perspective.

### Background

Germany, while having a highly developed genetic diagnostic and research sector, has a rather strict law for medical genetic diagnostics (Orth et al., 2011). Tests for medical purposes may only be carried out by specialized physicians and before obtaining informed consent they must inform clients about the nature, significance, extent, and possible consequences of the genetic test (so-called mandatory pre-test genetic counseling). The goal of this legal rule is to prevent possible harms and strengthen self-determination of the clients. In Germany there are, for example, app. 3,100 women seeking predictive genetic testing for BC each year,^2^ and there is app. 40,000 cases of genetic counseling focusing on predisposition for disease.^3^ However, the topics of risk and uncertainty concerning predictive genetic tests are almost unexplored in bioethical studies in Germany. But there is a variety of general literature about risk and uncertainty in the context of medical decision-making and genetic testing, and how they are perceived. In the following we will give an introduction to the main topics dealt with in the literature.

While uncertainty in the context of decision-making means that the outcomes of a decision or a future event are not known, the term “risk” specifically refers to potential negative outcomes or events. Uncertainty is a complex issue: Han et al. (2011) define it as the subjective consciousness of ignorance (“not knowing that one does not know”), while Gigerenzer (2007) distinguishes two different meanings of “uncertainty”: On the one hand, there are instances of *aleatory* uncertainty, referring to the natural unpredictability of future events. On the other hand, there is *epistemic* uncertainty, referring to the lack of knowledge regarding the accuracy of a risk prediction or its applicability to a specific case. Both are also relevant when handling predictive genetic information (Han et al., 2013). There is some evidence that lay people assess genetic risk information differently than professionals: For experts, such information is statistical data opening a window for rational decision-making, even though several studies have pointed out physicians’ inability to properly interpret genetic risk information and also their expressed reservations about their own understanding of and confidence in genetic risk assessment (Bodemer and Gaissmaier, 2012; Pachur et al., 2013; Falahee et al., 2017). However, it remains a crucial issue to what extent uncertainty has implications for lay people’s understanding of predictive genetic tests and decision-making and it has been suggested that lay people have a tendency to use stereotypes to make sense of the information (e.g., what we call in the results section of this paper the “50% rule”) (Gigerenzer, 2007; Pachur et al., 2013).

Lay people’s perception, however, is shaped by their own life circumstances, their experience of disease, their attitudes and beliefs, and their psycho-social situations (Johnson and Slovic, 1995; Fagerlin et al., 2007; Archibald and McClaren, 2013; Zikmund-Fischer, 2013; Rauscher et al., 2018). Thus, for lay people, probabilistic risk information is more a qualitative and intuitive idea rather than a quantitative, mathematical concept (e.g., Politi et al., 2007) – for them, *risk* remains a vague *uncertainty* that is often met with an ambivalent attitude (Wilde, 2009), and to which different conceptions are applied interchangeably (Sjöberg, 1999; Sorensen et al., 2008; Lipworth et al., 2010).

Two different understandings of risk used by lay people have been distinguished: (a) “risk as danger” in the sense that people associate the concept of risk with danger, i.e., the principal possibility of an adverse event; (b) “risk as a risk factor”: lay people often tend to understand risk in connection with risk factors such as an unhealthy diet, toxins or family history of disease, and point out the abstract nature of the idea of risk (Han et al., 2009; Engelhardt et al., 2017). In this sense, risk is used as a synonym or label for potentially health-damaging behaviors or external factors.

The main problem of these conceptual considerations is that there is no single “true” probability or “right” action for the individual patient and his/her future outcomes remain indeterminate and unknowable. Therefore, trust (or mistrust) in scientific expertise becomes highly relevant and it seems difficult for lay people to understand risk communication that focuses only on conveying the range of risks through numerical probabilities (Han et al., 2017). In addition, other studies show that lay people prefer numerical information in the context of medical risk information because they trust it more and feel more comfortable and satisfied with it than with vague estimates (Gurmankin et al., 2004; Bodemer and Gaissmaier, 2012). Moreover, the current practice of patient information is often biased toward a technical-rational approach and presents risk information in non-transparent formats (Bodemer and Gaissmaier, 2012). This indicates that uncertainty arises from limitations in the reliability, credibility, or adequacy of available information for lay people (Han et al., 2011).

The communication of genetic risks from physician to patient does not necessarily require a full understanding of all numerical information available. Lay people have different tolerance thresholds for genetic prognostic information and more often rely primarily on their already existing knowledge and beliefs, for example about the severity of diseases (Konrad, 2003; Bodemer and Gaissmaier, 2012; Damman et al., 2017). Taber et al. (2015) pointed out that lay people often perceive ambiguous genetic test results with more negative perceptions and that they tend to simplify them in stereotypical ways (e.g., “high” vs. “low” risk). This becomes problematic when individuals use their simplified misconstruction before healthcare providers have an opportunity to explain the principles of genetic testing to them and point out its complexity.

## Materials and Methods

We employed a qualitative approach in order to develop new hypotheses for future research on lay perspectives on and attitudes toward diagnostic possibilities (Bourgeault et al., 2011). We conducted seven FGs with lay people (*n* = 43) in four different German cities [Göttingen, Berlin, Frankfurt (Main), Cologne] in late 2016, thus receiving a broad range of societal backgrounds (Barbour, 2007). We developed a structured discussion guideline according to which, after a short thematic introduction, we applied case vignettes as a discussion stimulus (Barbour, 2007; Supplementary Material 1). Questions about the scenarios were designed to create a flow in the group discussions. The scenarios were modified during the discussion by adding further information and details as the discussion advanced. The FG guideline consisted of four realistic main scenarios, including predictive genetic testing for BC and early-onset AD, biomarker research for stratification in neo-adjuvant CoC therapy and the possibility of DTC GT, and whole genome sequencing (WGS).^4^ The themes of the vignettes were chosen to illustrate particular points. (a) BC was used as an example of a very common cancer disease with good treatment options and for which genetic screening programs are available in some countries. (b) In contrast, early onset AD was used as an example for a disease with no current cure options, which is associated with a strong stigmatization for patients and their relatives. (c) A genetic biomarker in CoC was used as an example of a treatment decision and treatment stratification. (d) The direct-to-consumer example was chosen to address questions about the utility and autonomy of an individual concerning his or her own genetic data. Each case vignette was supported with slides on which the risk information of the example was visualized (Schwartz and Woloshin, 2011; Supplementary Material 2). The participants received numerical risk information both verbally and visually by a graphic. In a pilot test, guideline and slides were pre-tested twice for improvement, with lay people and with academic staff. This test run allowed us to reduce redundancies and bias by wording, delete misunderstandings and improve the flow of discussion.

### Recruitment

In order to cover a large target group of lay people that are open for discussion and unprejudiced regarding the topics, we applied a mixed recruitment strategy using multiple channels and platforms. Participants were recruited via flyers and posters sent to public institutions (libraries, vocational schools, tech colleges, sports colleges) in the above-mentioned cities and in the above-mentioned cities local institutions providing genetic testing/counseling (genetic counseling practices). Additional recruitment was implemented online, using social media (e.g., Facebook), mailing lists (e.g., sports or nutrition groups) and small online ads (e.g., both digital and print and postings on virtual bulletin boards). If participants were interested in our study, they were asked for socio-demographic information (age, gender, educational background, profession, religion, marital status, number of children, daily internet use and previous experience with genetic testing). This information helped us to put together very socially diverse groups.

### Participants

Included participants were 18 years of age or older. Each group size varied between three and nine participants who were selected in terms of age, gender and educational background, including people with or without personal experience with genetic testing (Table 1; for a detailed description of the FG participant profiles and additional sociodemographic data see Supplementary Material 4).^5^ Informed consent was obtained from all individual participants included in the study.

**TABLE 1:** Socio-demographic data of the FG sample (*N* = 43) for each group.

Category	Specification	*N* (%)	FG I *n* = 7	FG II *n* = 8	FG III *n* = 5	FG IV *n* = 6	FG V *n* = 5	FG VI *n* = 9	FG VII *n* = 3
Sex	Females	26⁢(61)	5	5	4	5	3	2	2
	Males	17⁢(39)	2	3	1	1	2	7	1
Age	18–25	9⁢(21)	2	2	1	0	0	2	2
	26–35	14⁢(32)	3	3	1	3	1	3	1
	36–50	5⁢(12)	0	1	1	1	1	0	0
	51–70	11⁢(26)	2	1	2	2	2	2	0
	70+	4⁢(9)	0	1	0	0	1	2	0
Educational background	9 years	2⁢(5)	0	0	0	2	0	0	0
	10 years	4⁢(9)	0	1	1	0	1	1	0
	High school	11⁢(26)	1	3	1	1	0	3	2
	Vocational school	4⁢(9)	1	1	2	0	0	0	0
	Academic degree	22⁢(51)	5	3	1	3	4	5	1
Prior experience with genetic testing	Yes	12⁢(28)	3	3	1	2	0	2	1
	No	31⁢(72)	4	5	4	4	5	7	2

### Procedure

All participants were invited via e-mail and received a short pre-session info sheet superficially describing the general topics to be covered in the group discussion. This measure proved useful, as the discussion guideline was relatively extensive, leaving little room for questions. All discussions were audio-recorded for later transcription. Each group was moderated by two researchers (SiS, SW, MS). Furthermore, the researchers took additional minutes to reconstruct the discussion as an aid for transcription. All participants received an expense allowance of 25 Euro transferred into their bank account.

### Analysis

We conducted a qualitative content analysis (Bengtsson, 2016). The coding and analysis of the material was done by using the software Atlas.ti. All FG discussions were transcribed verbatim for the analysis. Transcripts were pseudonymized (Metschke and Wellbrock, 2002).^6^ We focused on comparing the statements in each group and the individual statements as well as the discussions’ flow and topics. The aim was to detect differences and similarities regarding attitudes and risk perceptions concerning genetic testing, with a special focus on dealing with numerical data. Our coding process was done in three different stages. First, we focused on an inductive coding with five basic codes and six subcodes (Supplementary Material 3). In the second stage we selected only the quotes in which we could find an explicit reference to numerical/statistical data. For the third stage we used deductive coding supplemented with the analysis of epistemic and aleatory uncertainty and focused on arguments related to numerical information. The third stage of coding revealed a strong co-occurrence (Contreras, 2011) of two codes for the evaluation of risk information: one coded risk information as a burden, the other as useful. This indicated an ambivalent perception of risk information as being a “useful burden.” The reporting of participants’ positions follows the scheme: many = > 50%, some = 10–50%, few = 0–10% of participants. We conducted peer-coding throughout the phase of analysis for the purpose of inter-coder reliability (Green and Thorogood, 2009).

## Results

Our overall analysis indicates that our participants showed a generally positive and open attitude toward predictive genetic testing. Especially young participants showed great interest in receiving extensive genetic information. However, apart from these general attitudes, it is notable how differently the provided examples of data on probability were interpreted. We found that numerical information (e.g., risk of 20% to get a specific disease) was interpreted and morally assessed in particular in connection with the factor of treatability; the more concrete treatment options were, the more positive the attitude of the participants toward predictive genetic testing. This way of assessing the information was based on four subtopics that emerged as dominant themes. We will discuss these in more detail in the following: Anumeric risk instead of statistical information; Treatment options as a factor for risk evaluation; Epistemic and aleatory uncertainty as moral criticism; Ambivalence as a sign of uncertainty.

### Anumeric Risk Instead of Statistical Information

Information on genetic risks, presented as numerical data (in absolute percent and visual graphics) in the context of *predictive genetic tests* (BC + AD) and *stratification* (e.g., biomarkers in the case of CoC), was interpreted by the participants in different ways. At first, participants hardly questioned these numbers. But in the following discussion, some participants criticized the use of probability figures as a means of support for decision-making, as they felt they lack the necessary abstraction capability. Many preferred a “50% rule” according to which figures above 50% were read as high. They also tried to fit figures <50% into hopeful schemes of interpretation, at least whenever the numbers were associated with an effective treatment option.

Mr. T. (FG5): But how serious is this diagnosis? […] There are also different degrees of leg fractures, right? And that’s just always the problem, how does a person deal with such an abstract probability of 50–60%. Well, that’s a little more than half. That’s not even that much. I’m in the other half. You can also calm yourself down very quickly, very easily, right? (Married, 51–70 years old, two kids, no experience with genetic testing).

#### Qualitative Binary Information Instead of Statistical Information

This is an example of a specific perception of numeric data that interprets the data in an optimistic manner if a concrete therapy and treatment option is available in the case of a disease; the numbers are then used as a reinforcement of a decision regarding intervention.

Ms. I. (FG2): So 71% is a nicer number for me than 15% (…) I also believe that this sort of numerical information can help defeat the cancer if you try to do it and stay strong. (Single, 18–25 years old, no kids, previous experience with genetic testing).

For this interpretation of the probabilistic data, diseases were classified as harmless (lifestyle, e.g., diabetes, being overweight) or severe (cancer); deadly (this information one should be certain about) or not deadly/treatable (this information is often disregarded) in order to counterbalance the complexity of decision-making. This argumentation can be seen as a confident strategy of justification by the participants, who, in turn, refer back to binary data systems (good/bad, high/low, small/big…).

The discussions about the different scenarios revealed how numerical information – also visually presented – was interpreted very subjectively.

Mrs. N. (FG2): I also think it’s a matter of type, one person says, good God, 55–65% probability is really not a little. Another person says, that’s not enough for me, I need 80, 90%, I don’t know. Difficult to say what, what one is to do, because it absolutely depends on everyone individually [and] on their life situation. How could you bear to know the risk and not do anything? (Married, 36–50 years old, two kids, no experience with genetic testing).

Referring to the use of a binary system, this quote serves as an example of how lay people preferred to convert statistical information into subjective – as we will call it – *anumerical* information about degrees of risk (“high,” “low,” “significant,” “enough,” etc.). The following discussion within the group about the BC scenario serves as a clear demonstration of this. In consideration of one’s own health and the need to take preventive action, the test was considered to be highly acceptable.

Life course and family responsibility was brought up as an argument to express favor toward the predictive test of BC:

Ms. H. (FG2): Yes, I mean, someday, it’s no longer just about yourself, you’re rather on your own while still young. That your parents outlive you is so far from (laughs) reality for you at that age, but someday, you’ll have a family, and someday, you’ll have responsibility, and you’ll also be part of a family you started yourself, and I think that really is a different situation then. […] I think it really does have something to do with age, maybe not necessarily only with the attitude of yours, which changes, but also with the situation you’re in […]. (Single, 26–35 years old, no kids, no experience with genetic testing).

This quote exemplifies that participants perceived a greater responsibility to their families when it comes to the risk of diseases affecting persons in a later stage their lives.

Overall, we found that participants in all FG had difficulties in dealing with the numeric information on risks provided in the predictive testing scenarios (for BC or AD). They considered the numerical data, which referred to future predictions of a disease, as too abstract. The future consequences of the numerical data for decision-making seem to be very complex, as this person’s statement illustrates:

Ms. F. (FG2): I find this decision very difficult. Or to have an opinion on that at all. I’m reluctant to deal with these kinds of topics much. Not because I’m not interested or want to keep my distance from diseases, but this idea of wanting to control everything, and to become a little obsessed with fears, and, to generally control life, I’m somewhat reluctant to do that. Because I also think it’s a fallacy. […]. For me, it would really only be an option if something very specific was brought to my attention. (Single, 26–35 years old, no kids, no experience with genetic testing).

#### Interpretation and Communication Is Delegated to Experts

Many participants expressed their discomfort with numerical information on risk and it became clear that they felt overburdened. Many refrained from positioning themselves in regard to the question whether they would consider a predictive genetic test for BC or AD risk in the case of a family history of the disease, and delegated the task to physicians.

Mr. D. (FG6): In that case I would ask myself: who will tell me about these results. […] If it was a doctor, we could talk about it. But… when we come to the question of which diseases I would want to know about and which ones I wouldn’t want to know about at all, I could say what I do not want to know about: diseases that could definitely break out in the course of my life. In which there is no probability, but absolute certainty. (Single, 18–25 years old, no kids, no experience with genetic testing).

#### Anumerical Information Used as a Stabilizing Element

*Anumerical* risks were often used as an argument for or against a genetic test of this type: If a disease has not yet developed (BC and AD), lay people used their experience-based knowledge of risks to cope with the unreliability of the information and to personalize it.

Ms. I. (FG2): Well, I think on the one hand, it’s frightening, and on the other hand in the case of such a result, you almost have to regret that you had the test carried out because it’s a rough estimate, it can happen but it doesn’t have to, a 50–50 chance, and if you don’t do the test and know that it runs in the family, you’ll also say, can happen, doesn’t have to happen. I’d actually prefer living with knowing that I have a 50–50% chance? I’d say that it’s more present at the back of your mind for the next 30 years than as if you simply didn’t take the test.

Mr. O.: But where’s the line, right? If you now say 80%, right.

Ms. I.: Yes, that’s true, of course. (Single, 18–25 years old, no kids, previous experience with genetic testing).

#### Numbers Used as a Reinforcement a Bridge

Thus, if a disease was very abstract and therefore scary (as it was the case with AD), the more difficult it was for participants to use the numbers as an assisting tool for their decision-making. Rather, these numbers led to an overall confusion.

Ms. C. (FG4): I wouldn’t want to know it in that case because I mean, it’s a disease you can’t treat yet. In the case of BC, there’s already something you can do. But as to AD I say, what use is that information to me? Can’t even change anything else, I mean, somehow. I mean, personally, I wouldn’t want that. (Partnership, 36–50 years old, one kid, no experience with genetic testing).

*Anumerical* risk information became relevant for the participants on different levels of decision-making. Initially, it represented a kind of analysis of the current situation as to how individual people emotionally react to test results. So the participants firstly wondered to what extent a test result of this disease might shock those affected. In addition, they expressed the worry that such a shock can inhibit the affected person to deal with the result in a rational way. The scenario examples of predictive results with a high probability of getting the disease were problematic especially for young people, because of their relation to the lifetime ahead of them. The expressed criticism revealed general insecurity about the disease where the internal perspective of those affected seems less accessible than for other diseases’ symptoms.

Mrs. N. (FG 2): If I knew, in my family, there are cases of AD, I wouldn’t want to know. I wouldn’t have a genetic test done either. Because in the case of BC, I would have had the chance to do something. Incurable means incurable. I’d just be scared […] The older you get the more you forget what you were about to do. Do I already have AD now? I wouldn’t want that. I’d want to accept it as symptoms of old age and experience it without constantly having to think about it. I’d know, of course, that it runs in the family. But I wouldn’t want it to be present. I’d just want to grow old in peace. And eventually, I’d become so forgetful. Then I wouldn’t have a test either… Especially since there’s nothing you can do. If there was an opportunity to do something… definitely, once medicine is advanced enough, then perhaps. (Married, 36–50 years old, two kids, previous experience with genetic testing).

Mrs. N. (FG2): In the case of AD, I mean, has a person with AD ever told me how he feels, what’s going on in his mind, which world he’s living in right now? It’s difficult, you don’t really know anything about it. (Married, 36–50 years old, two kids, no experience with genetic testing).

The FGs revealed various interpretations of AD. They all were taken from everyday life experience and not from medicine. AD and its implications are dealt with on an entirely hypothetical level, whereas in the section on BC the issue of prevention was discussed extensively. The arguments for or against a genetic prediction of dementia did not play a significant role, as there is no treatment option. Individual and family responsibilities played an important role, rather than the dealing with numbers and whether the predictive tests could better meeting those responsibilities.

### Treatment Options as a Factor for Risk Evaluation

In all FGs the benefits of advance-planning through the test for AD and therefore the advantage of early testing were discussed:

Mr. E. (FG1): Personally, I would have the test done. Also for the reason that if I know in a year, I have Alzheimer’s. Then I would focus my life very much (smiles) on that year again. Of course doing some preparations so that especially my relatives […] can start processing that and maybe take a few steps. Or that I myself am still able to, make preparations. That’s one side but I would in any case live my life differently when I know something like that. And given that point I would like to know if I don’t know anything anymore in a year. Until then I would really enjoy life again, I think. (Single, 26–35 years old, no kids, no experience with genetic testing).

Some participants found predictive tests only acceptable if they provided them with clear results, which can, above all, then give them clear options for their decision-making. It was important how well one could deal with uncertainty.

Ms. G. (FG6): Healing is an important aspect […]. So with BC it is likely, then I get the probability, but don’t know the consequences yet. If, as a result, BC really occurs, I will get sick, then… sometimes highly invasive interventions follow that significantly reduce the quality of life […]. An early form of Alzheimer’s… then the quality of life is impaired as well, and also to a degree in which especially the social environment is involved. Every social environment, too, is clearly affected just as it is with BC. (Life-partnership, 26–35, no kids, experienced with genetic testing).

Interestingly, in the case of clear medical treatment options, the major participants’ reaction in the stratification scenario (CoC) as well as the BC scenario was in favor of testing. In these scenarios, participants found the numerical information important, because they felt that the numbers made a decision for or against treatment easier. The information on risks continued to be interpreted in an optimistic way after the function of a stratification marker was explained to them. Participants saw the results as an “intervention” kind of treatment option with the aim of curing this disease. In the case of an early test result about AD participants saw as a chance for taking up further options of advance care planning, e.g., fitness, nutrition, etc.

Mr. O. (FG2): Well, I’d definitely have this done too. Well, only last year, I had a brother who developed CoC. But not that severe, so there was no pre-treatment necessary. But he also had to undergo chemotherapy then and all that. And actually tolerated everything quite well then, I think […]. I wouldn’t link it so much to the percentage now. I don’t know. I don’t know what I’m supposed to do with the percentage, whether it’s really exactly correct now. Okay, that’s a matter of statistics now, or calculating, but I’d also have it done in the case of 10% or so, because I think it also depends on the person himself. (Married, 51–70 years old, no kids, experienced with genetic testing).

#### Small Numbers Perceived as Glimmer of Hope/Denial of the Concept of Stratification

In most cases, the participants stressed the severity of the given cancer and insisted that every chance to fight it should be seized. The lower chance (35%) that a patient will respond well to therapy presented in the CoC scenario was not regarded as “small”; instead, it was seen as a “glimmer of hope” to justify deciding in favor of further testing and treatment. Participants assumed a connection between “low” percentages (35%) (the probability that a patient will respond well to therapy) and side effects, e.g., pain caused by therapy, and related this to own experiences and the experience of close relatives or friends who have received cancer therapy in the past. Therefore uncertainty has shown very clear in this scenario, as all participants denied the stratification impact associated with the biomarker. From their experience-based knowledge of cancer, every patient would always have chosen treatment. The utility of a treatment stratification based on improvement of quality of life (because without treatment no side effects occur) was only discussed by a small number of participants.

Another line of argumentation in the biomarker scenario was the idea that the result prompts the patient’s personal responsibility and intensifies one’s self-management competence regarding one’s own health.

### Epistemic and Aleatory Uncertainty as Moral Criticism

So far, we showed that there are morally relevant implications that influence whether or not participants want to know predictive genetic information. The following results deal with *why* one does not want to know any predictive test results. Overall, lay people are aware of epistemic and aleatory uncertainty, even though they do not use such terms. Epistemic uncertainty produced moral uncertainty in such a way that participants were unsure what is morally right in the sense that they wondered whether there can be a morally correct decision at all.

#### Reliability, Validity of Data Is Doubted

The participants often tended to not want to have predictive genetic information because of their lack of knowledge regarding the accuracy of risk predictions and their applicability to a particular case.

That is why many participants criticized the statement on probability in the different scenarios by making clear that it remains unclear how such a figure is supposed to help lay people to make a decision. Attempts to interpret the numbers fail due to the presumption that one is not a part of the statistic when dealing with the respective numbers.

Ms. I. (FG2): Well, I think on the one hand it’s frightening and on the other hand, that might sound stupid now, but in the case of such a result, you almost have to regret that you had the test carried out because it’s a rough estimate, it can happen but it doesn’t have to, a 50–50 chance, and if you don’t do the test, know that it runs in the family, haven’t done the test, then you’ll also say, can happen, doesn’t have to happen. I’d actually prefer living with what will be to knowing I have a 55% chance. Well, personally, I’d say that it’s more present at the back of your mind for the next 30 years than as if you simply didn’t take the test and it falls significantly further back. (Single, 18–25 years old, no kids, previous experience with genetic testing).

The quote illustrates an epistemic uncertainty that has been expressed in the discussions by many participants. The main question here was whether the information is reliable and applicable to the individual case, i.e., whether a predisposition comes into effect in the future or not:

Ms. S. (FG3): My first question is how is the average risk calculated in that sense? I mean is it supposed to show the average risk in the population? Ok and how can you know exactly that the average risk is 36.9%, is that from the participants who also did that? Or how do you want to know that for the whole population? That is my question anyway, because then you can never compare it like that. (Single, 18–25 years old, no kids, no experience with genetic testing).

#### Rejecting Idea of Control Over Life Course

Aleatory uncertainty was occasionally present in participants’ reactions to these discussions. Various statements emphasized it is not possible to predict a future event or not desirable to try. Participants rejected the idea of control without reasonable suspicion (from a physician) and believed that it produces more uncertainty than it would be helpful.

Ms. H. (FG 2): Well, I think I would at least need medical suspicion of an actual genetic problem. I’m convinced that, if you have such a test carried out, you’ll always find something. And there are cases like BC for example, where you carry it around like a burden all the time. Because you know it anyway. And because you see it in the family, which I think is a completely different thing than to simply undergo tests for any hereditary diseases even though none actually occur in the family, or for the risk of having a heart attack. We don’t have to know everything now. And I don’t think it increases the quality of life if you know you have a 10% risk of having a heart attack, um, at the age of 56. (Single, 26–35, no kids, no experience with genetic testing).

Ms. F. (FG2): I find this decision very difficult. Or to have an opinion on that at all. […] I’m reluctant to deal with these kinds of topics much. Not because […] I’m not interested or want to keep my distance from diseases, but this idea of wanting to control everything, and also to become a little obsessed with fears, and to generally control life, I’m somewhat reluctant to do that. Because I also think it’s a fallacy. (Single, 26–35 years old, no kids, no experience with genetic testing).

This thought resembles the idea of breaking down uncertainty to a binary option. Either event X will happen or it will not.

Mr. L. (FG7): I also think that it will burden her enormously because I think she also expected that, if a result comes, that it will be about 5–10%, and 55–65 simply are more than 50%. And if I think about roulette now, there’s black and red, right? You only have two variants. (Single, 18–25 years old, no kids, no experience with genetic testing).

This argumentation thus supports a criticism that genetic data does not provide control over the future.

#### Using Metaphors

Another aspect was pointed out by some participants who used metaphors like “scrying” or “lottery” to accentuate the difficulty of the genetic data.

Mr. T. (FG5): But it’s still relatively much crystal ball. It’s all just trends and tendencies. Which are in, in the genome. (Married, 51–70 years old, two kids, no experience with genetic testing).

By comparing a predictive genetic test with “esoterics” like fortune telling or scrying, some participants expressed their concerns about whether the data obtained by genetic testing and their interpretation would have a benefit for lay people and whether it is really reliable. This comparison can also comprise moral criticism of the unauthorized research for secrets.

The following quote illustrates once more the critical appraisal that predictive test results have insufficient evidence to conclude an individual genetic risk for a specific disease:

Mrs. J. (FG5): I don’t approve of that […] because I think, especially at the age of thirty, life starts to get exciting. And it really loses the excitement for me […] because I would only tick off then, does it occur, is it what was said there … how did it occur now? I mean, how did it come true or not come true? That’s as if I go to a woman who reads my fortune from a ball. I’d be constantly scared afterward whether she really hit the mark now or not. And here, it’s again […] completely different, there, it’s scientifically determined, justified. (Widowed, 70+ years old, three kids, no experience with genetic testing).

According to this statement, genetic tests are akin to astrology, regarded as producing no more than entertaining horoscopes, at best. It also shows the negative assessment that such data is not objective, but rather a subjective use of this data according to the slogan: if I believe in it, then it will also help me. This interpretation justifies the criticism that genetic information can potentially be harmful.

### Ambivalence as a Sign of Uncertainty

Interpreting probabilistic genetic test results made lay people feel insecure on an epistemic as well as a moral level. Participants expressed a notably ambivalent attitude toward predictive genetic tests which became apparent through many participants’ structure of argumentation. Ambivalence means that both supportive (e.g., it is useful, has benefits) as well as critical aspects (e.g., it is harmful) were inherent to many statements at the same time.

All group discussions of the different predictive scenarios started out accepting genetic testing. Consequently, participants tried to use the results from our scenarios as a source of information and were unsettled because of their lack of interpretability. In order to gain certainty for this unknown decision situation, they referred to already known rules for similar medical decisions. Thus, intuition or everyday experiences (which contain these rules) were used to arrive at a decision.

#### Reflecting the Consequences Before Performing a Genetic Test

Many argued based on this procedure. It arises because of participants’ “hidden” expectation of negative results (i.e., that the result will mean that they have a disease): This could be an effect of a culturally shaped expectation, since in the current medical system in Germany physicians are only able to prescribe genetic tests if there is a medical indication (e.g., family history or symptoms of a genetic disease). Therefore, ambivalence often came to light when numerical risks were related to one’s individual situation. This situation was feared to result in psycho-social problems when facing the results.

Ms. H. (FG2): Well, I’m just wondering, in the case that it’s confirmed that there’s an elevated risk of BC before the age of seventy, doesn’t that put you under even more pressure? Because that’s a long time span. And […] there’s still the risk that it won’t be detected, and I wonder whether my whole life would then […] revolve around me waiting for it to come. […] I think it’s a very good option if you then get the result that you don’t have this elevated risk but I wonder whether the fact that you know you have an elevated risk makes it worse than it was before. (Single, 26–35 years old, no kids, no experience with genetic testing).

The quote indicates personal concerns about dealing with a possible status of a “pre-patient.” One solution to this problem is to reflect the consequences before testing.

Mr. L. (FG7): I think I just have to be aware beforehand that this result can also have a negative result. I mean, it can also have a negative impact on me. And I simply have to be aware of that beforehand and I think I’d still be […] grateful to have so much information at once. (Single, 18–25 years old, no kids, no experience with genetic testing).

#### Regulation of Handling Genetic Risk Information Is Needed

Additionally, there is a noticeable ambivalence between the possibilities of receiving information versus the right to refuse being informed. Participants see their right not to know at risk in the case where they refuse to obtain information that might be cost-saving:

Ms. K. (FG1): And another point is also a matter of cost, because I find it dangerous that one could eventually say well, you knew that you have the risk, why didn’t you go to the check-ups. Now you have the disease and cause us costs. You are to blame for causing these costs now. (Life-partnership, 26–35, no kids, no experience with genetic testing).

This quote illustrates that there is a perception that genetic risk information is a special kind of information that needs regulation to prevent misuse to the detriment of individuals, but that also has far-reaching social consequences. A common significant point of criticism against genetic information lies in potential feelings of guilt of having passed on a disease to the next generation without knowing it:

Mrs. U. (FG3): Exactly, and if you can treat that or if you recover. So otherwise it is not worth it, right? So it is pointless, when I know that I have inherited a disease but I can’t do anything about it. (Married, 36–50, five kids, experienced with genetic testing).

#### Lack of Capability to Interpret Information Could Be a Burden

Nevertheless, many participants consider genetic tests to be useful because genetic data becomes relevant and interpretable in the form of numbers, even if only through knowledgeable experts (hence only feasible in combination with the classic doctor-patient relationship). An objection raised by many participants, however, is that the amount and complexity of information can place a burden on the users of genetic tests.

Ms. K. (FG1): And then I think it’s very important that you definitely have a good doctor with you […] with whom you have a trustful relationship. Who can easily explain these numbers. Because you know yourself there, I mean, well. Statistics is such a thing anyway, for many. And that you just have a good doctor who explains that to you or even get other opinions, from other doctors. (Life-partnership, 26–35, no kids, no experience with genetic testing).

These are important findings because in this context other participants argue that mobilizing their own social network is of the highest priority instead of, e.g., consulting a doctor and asking for his/her opinion. Then again, genetic tests are rejected when there are too few treatment options or they do not lead to better changes of curing a disease.

It becomes clear that this ambivalence results from bringing together the individual and the social perspective. Ambivalence is therefore not a phenomenon that affects only individual participants, but it is an important phenomenon in dealing with genetic risk information. In Table 2 we summarize the main results.

**TABLE 2:** Summary of the empirical results.

Result	Moral/social dimension
**Anumerical risk instead of statistical information**	

- Qualitative binary information instead of statistical information	- Self-determination
	- Responsibility – individual

- Interpretation and communication of genetic test results is delegated to experts	- Right to know
	- Trust in experts
	- Physician-patient-relationship

- Anumerical information is used as a stabilizing element	- Cope with unreliability
	- Utility of the results
	- Self-determination

- Numbers used as a reinforcement a bridge when making a decision regarding intervention	- Self-determination
	- Right to know

**Treatment options as moral factor**	

- Small numbers perceived as glimmer of hope/denial of the concept of stratification	- Utility of the results
	- Patients’ personal responsibility
	- Utility of the results
	- Mislead construct of responsibility

**Epistemic and aleatory uncertainty**	

- Reliability, validity of data is doubted	- (Mis-)Trust in medical experts

- Rejecting idea of control over life course	- Fate as relief
	- Reject idea of self-responsibility

- Metaphors like “scrying” or “lottery”	- (Mis-)Trust in results
	- (Mis-)Trust of the utility in using numerical results from predictive test

**Ambivalence as a sign of uncertainty**	

- Reflecting the consequences before performing a genetic test	- Self-responsibility
	- Self-determination
	- Trust in medical experts

- Need for regulation of handling genetic risk information (guilt because of a hereditary disease; receiving information versus don’t want to know)	- Responsibility of medical experts
	- Responsibility to the individual/family
	- Disadvantage of an individual regarding understanding genetic risk information
	- Fear of discrimination
	- Blaming for causing costs
	- Right to know or right not to know

- Lack of capability to interpret information could be a burden	- Responsibility of medical experts
	- Self-determination

## Discussion

In our study we showed that German lay people consider genetic testing as useful, but it also revealed a great deal of discomfort. Our results demonstrate that the perception of predictive genetic test results from lay people shifts when they try focusing on the perspective of a patient. Lay people argue from a healthy person’s perspective and therefore they are more likely to deal with the probabilistic forecasts than actual patients.

Similar to Viberg Johansson et al. (2017) we found that lay people tend to take genetic risk as a binary concept: you are at risk or you are not. Although we see in our results the division into binary codes, too, we observed the use of binary codes to simplify the weighing of the future consequences of a decision. Therefore the binary codes are more subtle, such as “high” vs. “low” or helpful (treatment) vs. harmful. This binary classification as well as the use of subjective experience (Hallowell, 1999; Han, 2013) helps lay people to interpret the complexity of risk information, but also to find a way to take responsibility for a risk decision. Our results show that for lay people dealing with the individual and family responsibilities plays a more important role than handling numbers.

Similar to other studies (e.g., Engelhardt et al., 2017), the current study indicates that genetic risk information is not only framed by statistical heuristics or numerical (il)literacy, but also strongly influenced by moral uncertainties and ambivalences. It should be pointed out here that people in everyday life do not (and perhaps cannot) optimize their decisions by calculating possibilities. Instead, they are much more likely to use every day heuristics that give them quick guidance rules for making decisions under uncertain conditions (Han et al., 2013).

Our findings also show that the anticipation of negative future emotions such as fear or regret results in criticisms of risk prediction (Schicktanz, 2017). Risk perceptions and subsequent decisions of people are highly influenced by their perceptions of personal/family risk factors, the experience of symptoms as well as by negative feelings such as fear of pain caused by individual experiences in dealing with illness (Konrad, 2010). However, appeals to people’s fears that do not inform about options for risk reduction may be counterproductive and may inhibit behavior change (Bandura, 2004). This was particular present in the discussion of the AD scenario, and it shows the ethical predicament patients are confronted with: to be able to use diagnostics for diseases for which there are no medical treatment options. Hence, lay people’s preferences depend on whether non-medical measures such as advance care planning are seen as valuable and meaningful. Apart from the ambivalence of an autonomous decision, it appears very obvious that participants felt helpless interpreting genetic risk information. It seems therefore problematic that by labeling individuals as “at-risk” and presenting genetic risks as manageable, genetic counseling implicitly places individuals under an obligation to attempt to modify these risks (Hallowell et al., 2003). Although many participants initially thought that genetic risk information was useful, many also see the development of an individual sense of “genetic responsibility” as rather problematic. For them, a critical reflection on the *why* of a genetic test and personal consequences of its results is the important theme. The perceived genetic risk also belongs to a lived experience of being affected. This also includes the experience of a perceived vulnerability of relationships, e.g., belonging to a group (family). The actual involvement probably generates a cognitive difference and forms a strong motivation to obtain information and to use genetic information (Raz and Schicktanz, 2009).

A significant finding was that participants transformed numerical risk information into subjective dimensions of highly morally loaded anumerical risk. When they felt uncertainty about the reliability or the psycho-social consequences, moral uncertainty and ambivalence were explicitly expressed. It is well known that heuristics or pre-existing knowledge are used for risk judgments (Kahneman and Tversky, 1972). Therefore, without evidence of real benefit and without expert advice that can adequately deal with the complexity and uncertainties of translating genetic data into individual health risks, it may be impossible for individuals to make informed enquiries and provide guidance.

We see the great importance attached to treatment options in our FGs, which also includes non-medical measures in the sense of advance care planning. This points at a connection to self-efficacy. From psychological theories we know that self-efficacy is a central determinant, as it influences the health behavior both directly and indirectly. Self-efficacy beliefs shape the results people expect their efforts to produce. Those with high efficacy expect them to develop positively. Those with low efficacy expect their efforts to yield poor results (Bandura, 2004). Therefore, the strong importance attached to the treatment options should always be considered and discussed against the background of self-efficacy.

Within the communication of “genetic risk” in our FGs participants refer to an *individual* and a *relational* conception of “genetic risk.” Lay people thus formulate their own embodied concerns as well as the embodied concerns of others (relatives or society) to whom they feel they belong and whom they feel obliged to care for (D’Agincourt-Canning, 2006; Lock, 2009). When they use metaphors like “read from the glass ball” or “fortune telling” to argue against the value of predictive genetic testing, they are addressing the responsibility of medicine (or society) in order to restore their socio-moral beliefs regarding their uncertain decision-making responsibility. Similar to Konrad (2005), we interpret this as a way of making genetic (pre-)knowledge an oracle in order to make the resulting “moral systems of foreknowledge” usable for everyday use in making decisions. This “moral system of foreknowledge” includes a pragmatic handling of uncertainty, which permeates the everyday lives of people with genetic predictive knowledge has an influence on whether and how to inform themselves and their social environment. The perceived expression of hope is not simply an emotion or a propositional attitude but resembles a virtue. Hope, expressed in metaphors, is an expression of despair caused by uncertainty of the probabilistic genetic information, and there seems to be a moral attitude not to give up. From this it can be concluded that there is a sense in which a “right to know” and the assumption of individual autonomy with respect to decision-making in connection with genetics can be problematic.

We found in our study that lay people often refer to epistemic uncertainty in their reasoning. Consistent with our findings are findings from the patients’ side: lay people often seem to realize the unpredictability of single events, yet probabilistic information is important for them across varying education and numeracy levels (Engelhardt et al., 2017).

Therefore many participants struggled with information about uncertainty. This especially applies to the imprecision of risk estimates (epistemic uncertainty), but also to the fundamental inability to predict individual futures (aleatory uncertainty). Particularly evident is lay people’s immense demand of support in dealing with predictive risk information. Risk here is more interpreted as a danger which appeals to a kind of genetic responsibility (Schicktanz, 2018). There are no qualitative results showing that lay people use strategies like trying to fully understand the information without being overwhelmed with complex statistical data using “optimistic thinking,” or the element of denial, in dealing with the predictive information in risk communication (Engelhardt et al., 2017). Ethically interesting here is that improved training of genetic counselors for better communication skills is often proposed by experts to address this communication problem (Han et al., 2017). Our results suggest that physicians/genetic counselors should not only present complex risk information in a simplified way, but also be sensitive to the counselee’s/patients’ simplification strategies and their motives. In addition, our results show that lay people tend to refer to *epistemic* uncertainty as a point of criticism. Such a criticism was primarily applied to the idea of genetic disorders and corresponds with scientific debates on the benefits of the previous predictive genetic predictions (Arribas-Ayllon et al., 2011b; Kohler et al., 2017).

From an ethical perspective, it is uncontroversial (UNESCO, 2004) that patients should be fully informed and should also be informed about the uncertain consequences arising on an individual and a social level when facing predictive genetic information. Failure to explicitly address epistemic and aleatory uncertainties may create misconceptions about the level of precision and individualization of probabilities presented during consultations (Tilburt et al., 2011).

This strategy once again points out that risk decisions are made in a relational context. Relational as an alternative approach to an individual autonomy approach referring to moral decisions involving the interests of others (Schicktanz, 2018). Responsibility here also includes moral social expectations toward the individual, combined with a strong sense of duty to share genetic risk information with potentially affected family members. Therefore, predictive genetic tests can lead to new options of acting but also affect intra-familial power relations. Therefore, predictive genetic test results should not only be seen as a case of privacy knowledge that creates new options for action but also affecting the balance of power within the family and in the physician-patient-relationship. The family is an important space in which the logics of risk have become entangled, incorporated and resisted (Arribas-Ayllon et al., 2011a). Regarding the family aspect we found a different picture of arguments toward responsibility. In order to be able to take responsibility, it has to be clarified who is responsible for whom. This in turn needs to be clarified on the basis of specific norms proved by specific instances and with specific consequences (Schicktanz and Schweda, 2012).

Personal experiences therefore seem to be key factors influencing screening decisions also for lay people (not only for patients) (Archibald and McClaren, 2013). Our results confirm that existing experience is very influential in shaping people’s perception of health and illness. When new information does not fit existing experiences, it does not have much priority for people and they become skeptical about it (Engelhardt et al., 2017). Moreover, genetic risk information is very abstract and genetic counseling is needed to help understand and deal with consequences of test results (Schaper et al., 2018). The skepticism is ethically problematic when the information does not lead to critical appraisal of risk but instead the information is optimistically misinterpreted to suppress its actual implications.

It can be concluded that providing lay people with probabilistic information might help to reduce insecurity in handling uncertainty – but not to eliminate uncertainty. It is a matter of debate whether such genetic test results should play a major role in the decision-making process (Hollands et al., 2016). Our results indicate that predictive genetic outcomes can turn into moral negotiations within and between family members that involve complex decisions about the (non-)disclosure of genetic information (the right not to know) about one’s own and others’ health care.

In the case of autonomy there are situations in which people may not have the capacity to deal with the information. Rantanen et al. (2008) argue that the counselor is expected to be able to also deal with these situations, but at the same time autonomous decision-making could not be guaranteed, since – and this is important in the context of consulting guidelines – the extent to which the principle of autonomy should be applied was not discussed in most guidelines. So while the current model of genetic counseling is described as non-directive, this standard might be ethically insufficient if more empowerment is needed for clients to make their judgments. Therefore empirical studies are needed to adopt and concretize such models.

Improving epistemic dimension of risk/uncertainty communication does not automatically solve moral problems related to control (e.g., self-determination or the right to know), but will at least avoid blurring lines between not-understanding and difficult decision-making.

According to important international guidelines and recommendations, qualified professionals are required for genetic counseling, and autonomous decision-making is an essential ethical requirement in decision-making regarding genetic testing (UNESCO, 2004; Rantanen et al., 2008).

A discussion on genetic counseling and its minimum ethical requirements for genetic counseling should account for the fact that predictive genetic tests will become increasingly common in the future. Counseling training should consider: (1) Psychological aspects of genetic risk information; (2) Lay people’s ambivalent perceptions of probabilistic information; (3) Ethical dimensions of predictive genetic tests. Finally, it is important to consider whether less autonomy-centered models of decision-making may be more suitable in particular cases (Louhiala and Launis, 2013). Perceptions of uncertainty affect people’s willingness or interest in having a test carried out as well as their interpretation of test results. This, in turn, is relevant for future improvement of genetic counseling practice.

### Limitations

A number of limitations pertain to this research. A first limitation is the use of the visual risk presentations in the scenarios. We could only provide a simplified version of a visual “decision aid” to present the risk information to our participants (Edwards et al., 2002). The visual presentation served only as an impulse to get the discussion started, the quality of this kind of visual presentation was not itself subject for discussion and evaluation (Schaper et al., 2018).

Second, as the method is qualitative in nature, we cannot claim representativeness of the group or generalize results for the broader population. Nevertheless, our large qualitative sample provides some heterogeneity. However, self-recruitment might have had an impact: more women than men took part in the FGs and people between 30 and 50 were underrepresented (the typical age of people working day jobs and are thus less likely to sacrifice free time for study participation). Similarly, other studies (Hallowell et al., 2006; Galarce et al., 2011; Dean, 2016) show a greater interest in health issues among women than among men. However, we could not find any explicit gender stereotypes or implicit gendered argumentations dominating the discussion as we found in other studies, where we examined organ donation issues, for example (Schicktanz et al., 2010).

Third, it is difficult to account for how individual personality traits may influence responses to genetic testing. How people deal with genetic information may depend on many different factors; this is not unique to information gained from genetic testing *per se*. Rather, we wanted to show in our study what role numerical understanding of probabilistic test results plays in dealing with genetic information.

Fourth, topics analyzed were also much broader than presented in this paper (incl. role of trust in medical systems as well as discussions of different forms of self- or family responsibility when dealing with genetic information). Due to the space limit, these topics will be discussed in other publications.

## Ethics Statement

This study was carried out in accordance with the recommendations of “University of Göttingen Human Research Review Committees Ref. Nr. 16/10/14,” with written informed consent from all subjects. All subjects gave written informed consent in accordance with the Declaration of Helsinki. The protocol was approved by the “University of Göttingen Human Research Review Committee.”

## Author Contributions

SW, MS, and SS contributed to the design of the work, analysis and interpretation of data, and drafting and revising the manuscript, approved the final version to be published, and agreed to be accountable for all aspects of the work.

## Conflict of Interest Statement

The authors declare that the research was conducted in the absence of any commercial or financial relationships that could be construed as a potential conflict of interest.

## Footnotes

1 We use “lay people” as a descriptive term for persons who are not experts in a particular field. However, we do not support the notion of a “deficit model” of lay people as it is prevalent in the more general discourse of public understanding of science (cf. Sturgis and Allum, 2004). Instead, we focus on the relevance of moral attitudes in handling genetic risk information.

2 https://www.vdek.com/presse/pressemitteilungen/2015/familiaerer-brustkrebs.html (accessed October 2, 2018).

3 https://www.gfhev.de/de/presse/pressemitteilungen/2005_bartram.htm (accessed October 2, 2018).

4 This article focuses on a detailed analysis of three scenarios, while the others are analyzed and published elsewhere [see Schaper et al., 2018 for the DTC GT part; Urban and Schweda, 2018 for the whole genome sequencing part; Wöhlke and Perry, unpublished (without DTC GT and WGS parts)]. This selection is due to the fact, that the different scenarios were designed to touch different ethical questions in the field of genetics and genomics. Naturally, the general discussion flow and each group dynamic were also considered for this focused analysis.

5 For a detailed methodological description (see Schaper et al., 2018). This table is already used first by Schaper et al. (2018).

6 In the following, all persons are randomly pseudonymized (Mr. A., Mrs. B., Ms. C., etc.), additional information about them is given in brackets after the respective quotes.

## Abbreviations

AD: Alzheimer’s disease
BC: breast cancer
CoC: colon cancer
DTC GT: direct-to-consumer genetic testing
FG: focus group.
