# Supplementary material for: How Uncertainty Influences Lay People’s Attitudes and Risk Perceptions Concerning Predictive Genetic Testing and Risk Communication
Source: Front Genet. 2019 Apr 26;10:380. doi: 10.3389/fgene.2019.00380 (PMC6497735; doi:10.3389/fgene.2019.00380)
Supplement: Supplementary file 1 [file Data_Sheet_1.docx]

**SUPPLEMENTARY MATERIAL 1**

Article: *How uncertainty influences lay people’s attitudes and risk perceptions concerning predictive genetic testing and risk communication*

Frontiers in Genetics, section ELSI in Science and Genetics

Authors: Sabine Wöhlke, Manuel Schaper, Silke Schicktanz

Department of Medical Ethics and History of Medicine, University Medical Center Göttingen, Germany

Correspondence: Dr. Sabine Wöhlke, [sabine.woehlke@medizin.uni-goettingen.de](mailto:sabine.woehlke@medizin.uni-goettingen.de)

THIS GUIDELINE CONTAINS AN ADDITIONAL HYPOTHETICAL SCENARIO WITH A DIFFERENT TOPICAL FOCUS ON WHOLE GENOME SEQUENCING, THAT WAS NOT ANALYZED FOR THIS ARTICLE

**The focus group discussion will be aided by the PowerPoint presentation ‘Genetic Testing Focus Group UoB Presentation’, referred to below as Slide 1, Slide 2, etc.**

**Information about the focus groups and consent / background questionnaires**

# Scenario 1: Genetic tests for the prediction of diseases

# Breast cancer

Let’s start with the first scenario. Imagine the following situation: Ms. Jones has a family history of breast cancer – if it is detected at an early stage breast cancer is often treatable. She could choose to have a genetic test carried out by her doctor to determine whether she has an increased risk of developing breast cancer before the age of 70.

1. What, in your opinion, could motivate Ms. Jones to have such a genetic test, what could deter her from doing so?
2. What kinds of additional information might someone need to decide whether they should have a genetic test carried out in this situation?

**Slide 4**

Now let’s imagine that Ms. Jones has decided to have the genetic test: The results show that her risk of developing breast cancer before the age of 70 is increased and turns out to be 55-65%. This means that 55-65 people out of every 100 people with this kind of genetic profile would develop the disease.

1. To what extent could the test results influence Ms. Jones’ well-being, her attitudes towards life and her behavior? [How might she feel if she needed to make changes to her lifestyle to try and reduce her risk of developing breast cancer?] [What kinds of additional information might she need to help her deal with this test result?]
2. Should Ms. Jones inform other people about the results of her genetic test? If yes, who (and who not) should she inform, and why? Would you inform other people about the results of this genetic test, if you were in a similar situation?
3. **Early-onset Alzheimer’s disease**

Now we’ll change the scenario a bit to a different disease: Imagine now that it’s not about the risk of developing breast cancer but about the risk of developing an early-onset form of Alzheimer’s disease. This disease is not yet treatable.

1. How would you feel about someone taking a genetic test for the purpose of predicting their risk of developing Alzheimer’s disease? [Would your previous viewpoint change? To what extent? Why?]

**Slide 5**

Imagine that Ms. Jones takes this genetic test and the results reveal that her risk of developing Alzheimer’s disease before the age of 65 is at 85%. This means that 85 out of every 100 people with this kind of genetic profile would develop the disease.

1. What are your feelings about this genetic test now? When faced with this kind of test result, would this change your opinion about the value of this kind of genetic test? How?

Thank you for your contributions so far!

**Scenario 2: Genetic tests to provide information for treatment decisions**

Now for a new scenario**:** Mr. Shah, aged 55, has colon cancer at an advanced stage. At such an advanced stage, standard treatment includes pre-treatment consisting of radiation and chemotherapy. This pre-treatment, which has side effects, could shrink his tumor and thus, reduce the severity of surgery he needs and increase his chances of survival. However such pre-treatment is not always effective and a genetic test could provide Mr. Shah and his doctors with information about how likely it is that radiation and chemotherapy would be effective for him.

1. What are your feelings about such a genetic test?

**Slide 7**

1. Mr. Shah’s genetic test result shows that there is a probability of 71% that he would benefit from pre-treatment with radiation and chemotherapy. This means that the treatment would work for 71 out of 100 people with the same genetic test results. How do you feel about whether Mr. Shah should opt for or against this treatment and why?

**Slide 8**

1. Now imagine instead that the genetic test result shows that there is a probability of 35% that Mr. Shah would benefit from pre-treatment with radiation and chemotherapy. This means that the treatment would work for 35 out of 100 people with the same genetic test result. Does this change your views about whether Mr. Shah should opt for or against this treatment? Why?

Thank you for your opinions so far!

# Scenario 3: Research by means of modern techniques of genome sequencing

We will leave Mr. Miller and will come to a new scenario. Imagine that Mr. Meier (30 years of age) is invited to participate in a medical trial, in which, among other things, his whole genome, meaning his complete genetic information, will be collected and analyzed. This means that very large amounts of genetic information are collected which can provide information about various predispositions and traits such as his blood type, ethnicity, intelligence or obesity but also about all kinds of diseases and disease risks.

1. What do you think about the possibility of obtaining such comprehensive information by means of whole genome sequencing?
2. To which extent would you want to be informed about the results of such an analysis (for example in the case of certain disorders)? Which kind of reports should you definitely be informed about (diseases – severe/not severe; treatable/untreatable)?
3. It is possible that, also in the future, new information can be obtained from Mr. Meier’s genomic data about previously unknown dispositions and disease risks, as research evolves and produces new insights about genetics, for example. To which extent should Mr. Meier be informed about such new information regarding prospective dispositions and results? And how could regulations be implemented regarding what and how much would be communicated at a later point in time?
4. In the context of the trial, Mr. Meier could also share his data for further use in future research projects. Under which circumstances does such sharing of data seem acceptable to you? [To which extent would it make a difference to you whether the respective projects take place at a public institution or at a company / domestically or abroad?]

Thank you for your arguments so far.

# Scenario 4: Direct-to-consumer genetic testing

Now let’s consider the final scenario: Genetic testing is not only carried out by doctors or in clinical settings or research trials, but in some countries people can buy a genetic test from a private company over the internet. This kind of testing is known as “direct-to-consumer” genetic testing. These tests can provide information about someone’s risk of developing a wide range of diseases, such as Alzheimer’s disease, cancer or diabetes, and can also give information about other genetic predispositions such as being overweight, or hair loss.

I can show you a couple of examples of the websites of companies that provide this kind of test…

**Slides 9-10**

- 1. What do you think about the availability of this kind of genetic test (Example 1: diseases and disorders; Example 2: life style test)? [Are there any kinds of genetic information that should be included or excluded from tests like this?]
  2. Would you be interested in ordering a genetic test like this? [Why? What would stop you from ordering a test like this?]
  3. Would you want such direct-to-consumer genetic tests to be legally prohibited in the United Kingdom?

**Slide 11**

- 1. Here you can see an example of a test result page from a direct-to-consumer provider: Imagine that these are your results: What do you think these results show about your risk of suffering from obesity, lung cancer or multiple sclerosis?
  2. What would you think about providing your genetic data to a company like this for research purposes?

**Slide 12**

On this example of such a test result some of the results are different (highlighted in red). What do you think these results show about your risk of developing these conditions now? [How would you feel if these were results? What would you do if these were your results?]

# Conclusion

# Slide 13

Thank you all so much, this has been a really fascinating and useful discussion. We have now come to the end of the session, is there anything else you would like to share or ask at this point?

Thank you very much for taking part, I hope you have a good journey home.
