# Supplementary material for: How Uncertainty Influences Lay People’s Attitudes and Risk Perceptions Concerning Predictive Genetic Testing and Risk Communication
Source: Front Genet. 2019 Apr 26;10:380. doi: 10.3389/fgene.2019.00380 (PMC6497735; doi:10.3389/fgene.2019.00380)
Supplement: Supplementary file 2 [file Data_Sheet_2.pdf]

SUPPLEMENTARY MATERIAL 2

Article: *How uncertainty influences lay people's attitudes and risk perceptions concerning predictive genetic testing and risk communication*
Frontiers in Genetics, section ELSI in Science and Genetics
Authors: Sabine Wöhlke, Manuel Schaper, Silke Schicktanz
Department of Medical Ethics and History of Medicine, University Medical Center Göttingen, Germany
Correspondence: Dr. Sabine Wöhlke, sabine.woehlke@medizin.uni-goettingen.de

UNIVERSITÄTSMEDIZIN GÖTTINGEN UMG

Focus Group Study

Lay perspectives and attitudes toward new possibilities in genetic diagnostics

What is genetic information?

Genetic information is information about hereditary traits, that is contained in every cell in form of the DNA. The sum of all genetic information contained in every cell is called the genome.

Genetic analysis allows to read parts of the genome that are responsible for specific traits. New technologies are able to read the whole genome, so that all genetic information is known.

Why genetic analysis?

Genetic analysis serves as a means to obtain genetic information for different purposes. In medicine, genetic testing plays an important role in two areas:

In *iagnostics*, genetic information is used to determine a disease, or the possibility that someone could inherit a certain disease.

In *prediction,* genetic information is used to predict, if someone will get a disease in the future.

Frau Wagner

Breast cancer in family

Genetic test:
Risk of 55-65% before age of 70 → Breast cancer

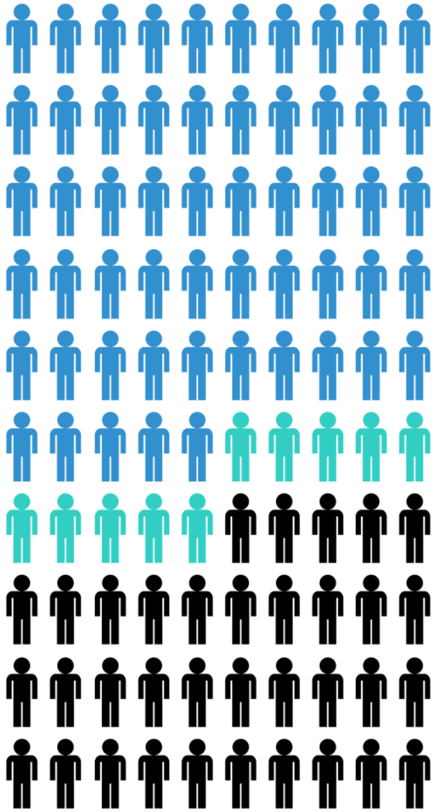

Frau Wagner

Early-onset Alzheimer in Family

Genetic test: Risk of 85% before age of 65
→ early-onset Alzheimer

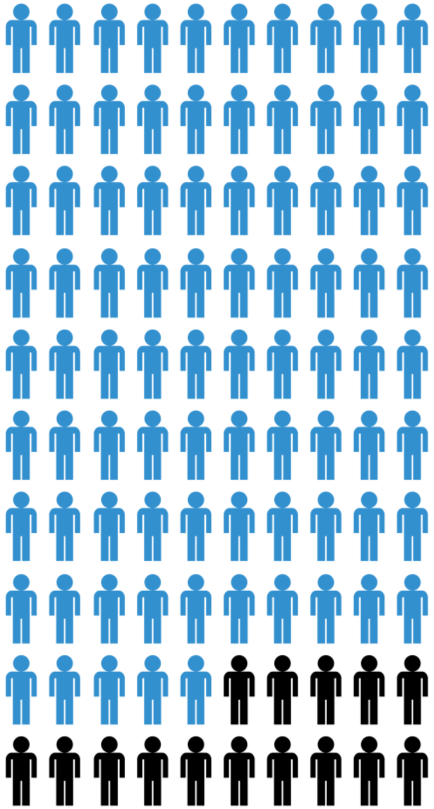

Herr Schmidt

Colon cancer

Genetic test: Chance of 71% to benefit of pre-treatment with radiation and chemotherapy

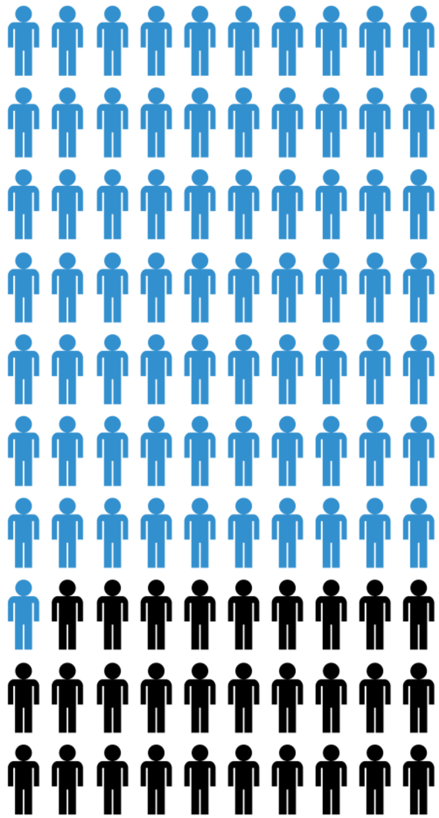

Herr Schmidt

Colon cancer

Genetic test: Chance of 35% to benefit of pre-treatment with radiation and chemotherapy

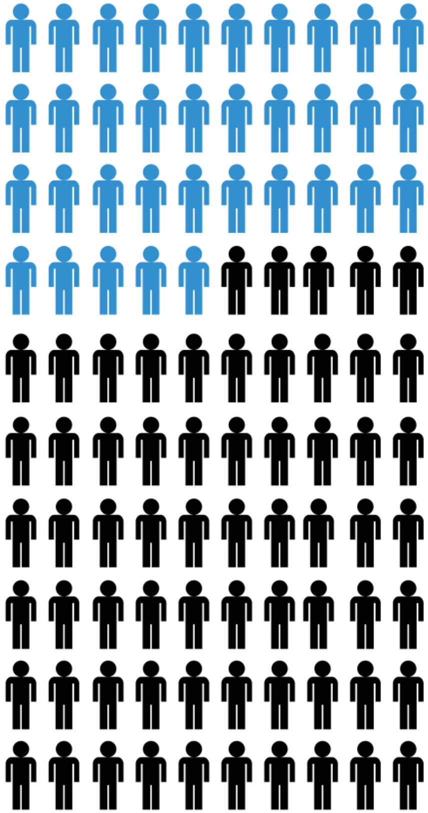

Herr Meier

Study participation
Complete analysis of genome

What information about results?

Genetic testing offered online (1)

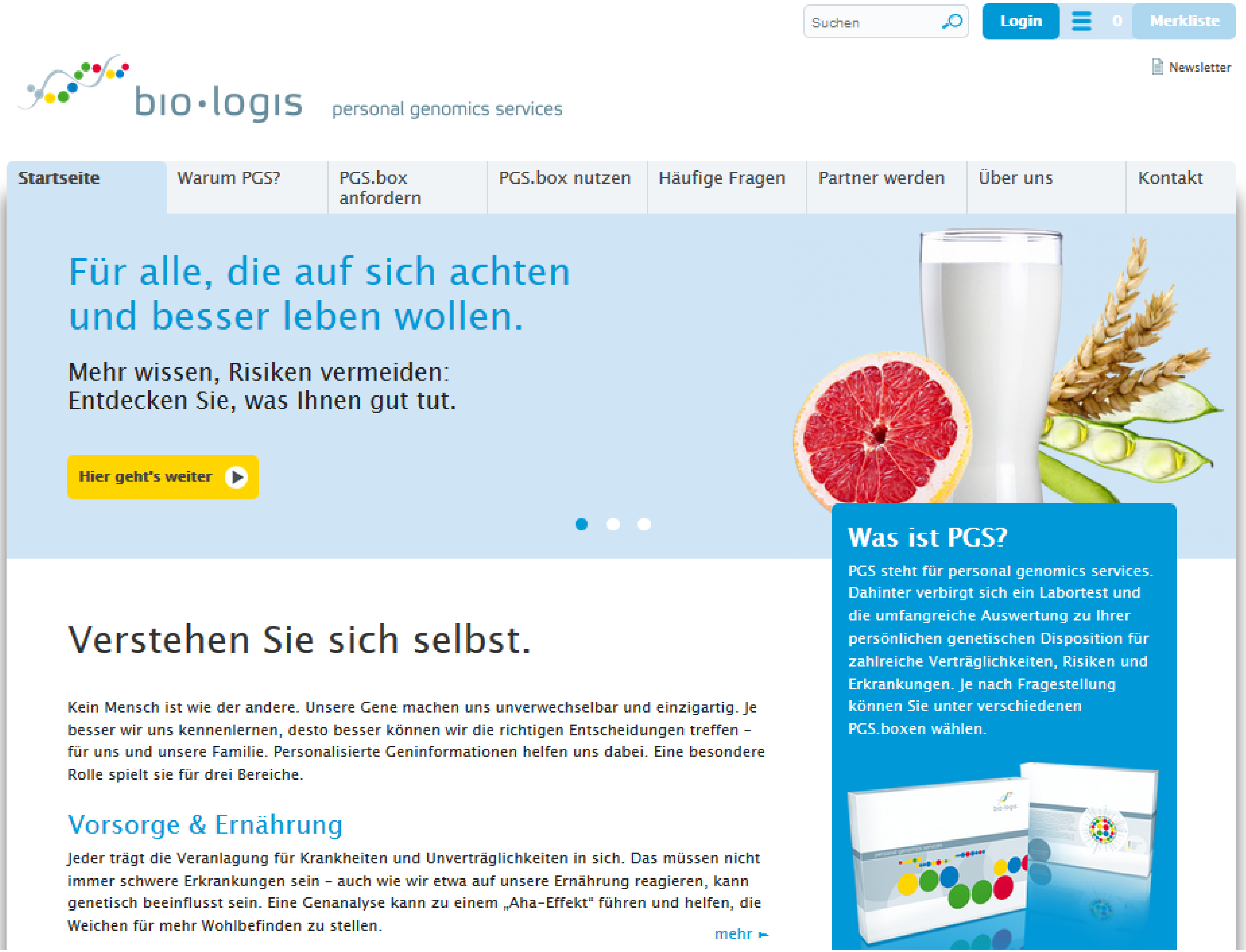

Genetic testing offered online (2)

Willkommen auf easyDNA.at

ISO 17025
Akkreditiertes Testen

Home
Kontaktieren Sie uns
Lassen Sie Uns Sie Anrufen
Hier Bestellen

VISA MasterCard PayPal

DNA-TESTS

Vaterschaftstest
Pränataler Vaterschaftstest
Beziehungstest
Untreuetest
Diskrete DNA Proben
Baby-Geschlechtstest
DNS-Gesundheitstest

OPTIONEN

Häufig Gestellte Fragen

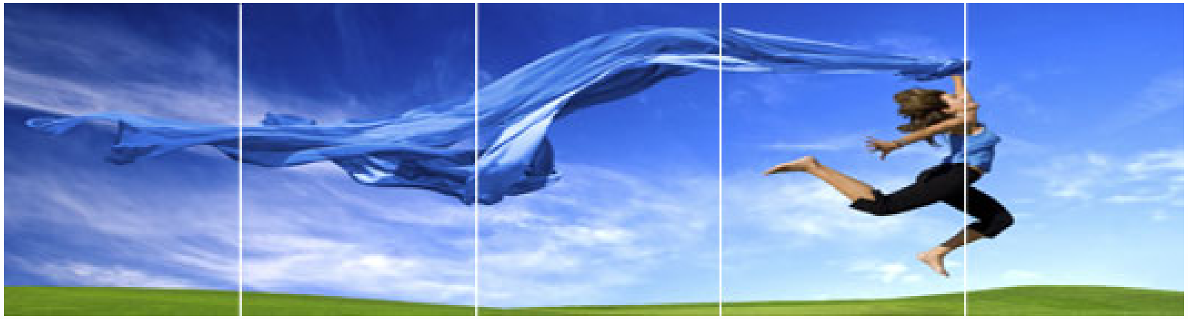

DNS-Gesundheitstest genetischer Veranlagung

Stellen Sie sich vor, was Sie tun könnten, wenn Sie die Möglichkeit hätten, herauszufinden, wie hoch Ihr Risiko ist, bestimmte Krankheiten und Erkrankungen zu entwickeln, indem Sie die Informationen in Ihrer DNS entschlüsseln. easyDNA Österreich bietet einen **innovativen genetischen Test** an, der Ihnen genau das ermöglicht; die Chance wahrzunehmen, selbst die Kontrolle über Ihre Gesundheit und Ihr Wohlbefinden zu übernehmen.

Der Gedanke ans Altern und das Risiko, eine Krankheit wie Krebs zu entwickeln, können angsteinflößend sein, durch den Einblick in ihren genetischen Code können Sie jedoch lernen, auf welche Zeichen Sie achten sollten und welche Vorsichtsmaßnahmen Sie einleiten sollten. Neueste Erkenntnisse in der Genetik von Autoimmunerkrankungen haben dies möglich gemacht.

Example of test result page of a test offered online: **Hr. Müller**

Name	Confidence	Your Risk	Avg. Risk
Obesity			63,9%
Coronary Heart Disease			46,8%
Atrial Fibrillation			27,2%
Psoriasis			11,4%
Lung Cancer			8,5%
Gallstones			7,0%
Chronic Kidney Disease			3,4%
Parkinson`s Disease			1,6%
Multiple Sclerosis			0,3%
Bipolar Disorder			[illegible],10%
Breast Cancer ♀			0,00%
Lupus (Systemic Lupus Erythematosus) ♀			0,00%

Obesity is defined as an increased

Coronary heart disease (CHD) is a disease in which a waxy substance called plaque builds up inside the coronary arteries that supply oxygen-rich blood to the heart. As a consequence the tissue is not sufficiently supplied with oxygen.

[illegible] ion of [illegible] ts and [illegible] ully [illegible] stem [illegible] ers to [illegible] thus belongs to the demyelinating diseases. As a consequente, patients develop paralyses and involuntary muscle contractions (spasms). What causes the autoimmune reaction is unclear, yet some viruses are suspected to play a role. Genetic and environmental factors seem to play a role in the development of Multiple Sclerosis.

[illegible] lupus erythematosus can affect all organs.

Example of a test report taken from a website: **Hr. Bauer**

Name	Confidence	Your Risk	Avg Risk
Obesity	★★★★	36,7%	63,9%
Coronary Heart Disease	★★★★	50,2%	46,8%
Atrial Fibrillation	★★★★	29,3%	27,2%
Psoriasis	★★★★	9,9%	11,4%
Lung Cancer	★★★★	6,9%	8,5%
Gallstones	★★★★	6,2%	7,0%
Chronic Kidney Disease	★★★★	3,4%	3,4%
Parkinson`s Disease	★★★★	10,4%	1,6%
Multiple Sclerosis	★★★★	0,4%	0,3%
Bipolar Disorder	★★★★	5,7 %	0,10%
Breast Cancer ♀	★★★	0,00%	0,00%
Lupus (Systemic Lupus Erythematosus) ♀	★★★	0,00%	0,00%

Thanks for your participation!
