# Supplementary material for: How Uncertainty Influences Lay People’s Attitudes and Risk Perceptions Concerning Predictive Genetic Testing and Risk Communication
Source: Front Genet. 2019 Apr 26;10:380. doi: 10.3389/fgene.2019.00380 (PMC6497735; doi:10.3389/fgene.2019.00380)
Supplement: Supplementary file 3 [file Data_Sheet_3.docx]

**SUPPLEMENTARY MATERIAL 3**

Article: *How uncertainty influences lay people’s attitudes and risk perceptions concerning predictive genetic testing and risk communication*

Frontiers in Genetics, section ELSI in Science and Genetics

Authors: Sabine Wöhlke, Manuel Schaper, Silke Schicktanz

Department of Medical Ethics and History of Medicine, University Medical Center Göttingen, Germany

Correspondence: Dr. Sabine Wöhlke, [sabine.woehlke@medizin.uni-goettingen.de](mailto:sabine.woehlke@medizin.uni-goettingen.de)

*Analysis: Main categories and coding list used in the analysis of focus group transcripts*

| **Code category** | **Code** | **Code description** |
| --- | --- | --- |
| **Risk information** | **Benefit_pro** | Risk information is attributed a certain benefit |
|  | **Benefit-**_**contra** | The benefits of risk information are seen critical |
|  | **Risk_information_sharing** | Participants are in favor or against sharing genetic data with third parties / institutions |
|  | **Risk_burden** | Risk information negatively affects well-being and quality of life |
|  | **Risk_hope** | Risk is associated with confidence; The will to stand up to the risk is expressed |
|  | **Risk_responsibility** | Risk information appeals to responsibility in a proactive sense |
| **Motivation** | **Attitude_planning** | Evaluation of genetic test regarding life planning |
|  | **Attitude_lifetime** | Evaluation of a genetic test regarding the burden of the test result and the remaining lifetime |
|  | **Attitude_result-dependent** | Ambiguous evaluation depending on yet unknown testing outcome |
| **Condition** | **Condition_ treatable** | Condition for the decision for a genetic test is the treatability or prevention options of a disease |
|  | **Condition_indication** | Condition for the decision for a genetic test is specific evidence (e.g., family history) |
| **Dealing with numbers / probabilities** | **Include numbers / probabilities** | Statement refers to numbers/ probabilities |
|  | **Include anumerical risk** | Statement is referring to risk that is not expressed in numbers, but as a possibility that is based on experience or observation. Comparisons, relationships are used and other factors such as age, life situation are included |
|  | **Lack of interpretability** | Interpretation of risk information overwhelms the participants |
|  | **Experiences based knowledge** | Statements that rely on own experience or everyday life knowledge |
| **Uncertainty** | **Aleatory uncertainty** | Statements that refer to the natural unpredictability of future events |
|  | **Epistemic uncertainty** | Statements that refer to lacking knowledge regarding the accuracy of a risk prediction or its applicability to a case |
