# Supplementary material for: How Uncertainty Influences Lay People’s Attitudes and Risk Perceptions Concerning Predictive Genetic Testing and Risk Communication
Source: Front Genet. 2019 Apr 26;10:380. doi: 10.3389/fgene.2019.00380 (PMC6497735; doi:10.3389/fgene.2019.00380)
Supplement: Supplementary file 4 [file Data_Sheet_4.docx]

**SUPPLEMENTARY MATERIAL 4:**

**FOCUS GROUP COMPOSITION AND PARTICIPANT PROFILES**

Article: *How uncertainty influences lay people’s attitudes and risk perceptions concerning predictive genetic testing and risk communication*

Frontiers in Genetics, section ELSI in Science and Genetics

Authors: Sabine Wöhlke, Manuel Schaper, Silke Schicktanz

Department of Medical Ethics and History of Medicine, University Medical Center Göttingen, Germany

Correspondence: Dr. Sabine Wöhlke, [sabine.woehlke@medizin.uni-goettingen.de](mailto:sabine.woehlke@medizin.uni-goettingen.de)

|  | **Pseudonym^^[[1]](#footnote-1)^^** | **Prior experience with genetic testing** | **Sex** | **Age** | **Educational background** | **Marital status** | **No. of kids** | **Religion** |
| --- | --- | --- | --- | --- | --- | --- | --- | --- |
| FG I | Ms. D. | Yes | f | 18-25 | High School | Single | 0 | None |
|  | Mrs. E. | Yes | f | 51-70 | Academic Degree | Divorced | 0 | None |
|  | Ms. L. | Yes | f | 18-25 | Academic Degree | Single | 0 | Muslim |
|  | Ms. K. | No | f | 26-35 | Academic Degree | Life-partnership | 0 | Catholic |
|  | Mr. E. | No | m | 26-35 | Academic Degree | Single | 0 | None |
|  | Mr. A. | No | m | 26-35 | Academic Degree | Single | 0 | Protestant |
|  | Mrs. M. | No | f | 51-70 | Vocational School | Divorced | 1 | Protestant |
| FG II | Ms. I. | Yes | f | 18-25 | Academic Degree | Single | 0 | Protestant |
|  | Mrs. P. | No | f | 70+ | High School | Widowed | 3 | Protestant |
|  | Ms. H. | No | f | 26-35 | Academic Degree | Single | 0 | Catholic |
|  | Mrs. N. | Yes | f | 36-50 | High School | Married | 2 | Catholic |
|  | Ms. F. | No | f | 26-35 | Academic Degree | Single | 0 | None |
|  | Mr. U. | No | m | 18-25 | High School | Single | 0 | None |
|  | Mr. O. | Yes | m | 51-70 | 10 years | Married | 0 | None |
|  | Mr. X. | No | m | 26-35 | Vocational School | Single | 0 | Protestant |
| FG III | Mr. Y. | No | m | 51-70 | Academic Degree | Divorced | 0 | None |
|  | Ms. S. | No | f | 18-25 | High School | Single | 0 | Catholic |
|  | Ms. Q. | No | f | 26-35 | Vocational School | Single | 0 | Orthodox |
|  | Mrs. U. | Yes | f | 36-50 | 10 years | Married | 5 | Muslim |
|  | Ms. Z. | No | f | 51-70 | Vocational School | Single | 0 | Buddhist |
| FG IV | Mr. B. | No | m | 26-35 | Academic Degree | Single | 0 | None |
|  | Mrs. T. | Yes | f | 26-35 | Academic Degree | Married | 3 | Jewish |
|  | Mrs. Y. | Yes | f | 51-70 | 9 years | Divorced | 1 | Catholic |
|  | Ms. A. | No | f | 26-35 | High School | Life-partnership | 0 | Catholic |
|  | Ms. C. | No | f | 36-50 | Academic Degree | Partner | 1 | None |
|  | Mrs. X | No | f | 51-70 | 9 years | Single | 0 | Catholic |
| FG V | Mrs. R. | No | f | 51-70 | Academic Degree | Widowed | 0 | None |
|  | Ms. W. | No | f | 36-50 | 10 years | Single | 2 | Protestant |
|  | Mr. W. | No | m | 26-35 | Academic Degree | Single | 0 | None |
|  | Mrs. J. | No | f | 70+ | Academic Degree | Widowed | 3 | None |
|  | Mr. T. | No | m | 51-70 | Academic Degree | Married | 2 | None |
| FG VI | Ms. G. | Yes | f | 26-35 | Academic Degree | Life-partnership | 0 | Protestant |
|  | M3. K. | No | m | 51-70 | Academic Degree | Married | 1 | Catholic |
|  | Mr. M. | No | m | 18-25 | High School | Single | 0 | Protestant |
|  | Mr. N. | No | m | 26-35 | Academic Degree | Married | 0 | Protestant |
|  | Mr. D. | No | m | 18-25 | High School | Life-partnership | 0 | Protestant |
|  | Mr. P. | No | m | 70+ | Academic Degree | Married | 2 | None |
|  | Mrs. O. | Yes | f | 26-35 | Academic Degree | Married | 0 | None |
|  | Mr. Q. | No | m | 70+ | 10 years | Divorced | 1 | None |
|  | Mr. G. | No | m | 51-70 | High School | Single | 0 | None |
| FG VII | Ms. V. | Yes | f | 18-25 | High School | Single | 0 | Protestant |
|  | Ms. B. | No | f | 36-50 | Academic Degree | Single | 0 | Catholic |
|  | Mr. L. | No | m | 18-25 | High School | Single | 0 | Protestant |

1. Letters may be assigned to two both a male and a female participant, not implying relationships or kinships. [↑](#footnote-ref-1)
